# Case Report: An incidentaloma that catches your eye - adrenal myelolipoma

**DOI:** 10.12688/f1000research.11766.1

**Published:** 2017-07-18

**Authors:** Rosanna D'Addosio, Joselyn Rojas, Valmore Bermúdez, Flor Ledesma, Kyle Hoedebecke

**Affiliations:** 1 Department of Public Health, School of Medicine, University of Zulia, Maracaibo, 4002, Venezuela; 2 Division of Pulmonary and Critical Care Medicine, Brigham and Women’s Hospital and Harvard Medical School, Boston, MA, 02115, USA; 3 Endocrine and Metabolic Diseases Research Center, School of Medicine, University of Zulia, Maracaibo, 4002, Venezuela; 4 Research group Altos Estudios de Frontera (ALEF), The Simón Bolívar University, Cúcuta, Colombia; 5 WONCA Polaris - USA, Bangkok, 10500, Thailand; 6 Yongsan Health Clinic, Seoul, 96205, Korea, South

**Keywords:** Myelolipoma adrenal, adrenal incidentaloma, benign adrenal tumor

## Abstract

**Background**: Adrenal incidentaloma refers to the incidental finding of a tumor in the adrenal gland, where nonfunctional forms are the most common variant. Myelolipoma is a rare (0.08-0.4%) occurrence characterized by adipose and hematopoietic tissue. The aim of this case report is to describe the diagnosis and appropriate management of a myelolipoma in an asymptomatic patient, which was originally considered an incidental hepatic hemangioma prior to being identified as a giant adrenal adenoma.

**Case description:** The patient was a 54 year old obese female with a recent diagnosis of diabetes type II and dyslipidemia with recent ultrasound imaging suggestive of a hepatic hemangioma. An MRI was performed revealing a 7x6cm lesion in the right adrenal area indicating a giant adrenal adenoma. An adrenalectomy was performed without complications. The pathology report identified a myelolipoma.

**Discussion:** The incidence of myelolipoma has recently increased due to advances in radiological techniques. Its etiology is unclear and the most accepted theories support a myeloid cell metaplasia in the embryonic stage as a result of stress, infections, or adrenocorticotropic hormone or erythropoietin stimulus. Contributing components may include bone morphogenetic protein 2 and β-catenin, as well as the presence of the chromosomal translocation (3, 21) (q25; p11). Despite its benign nature, the association with other adrenal lipomas must be ruled out. A biochemical evaluation is essential for detecting subclinical states, such as Cushing syndrome and pheochromocytoma.

**Conclusion:** Adrenal myelolipomas are rare benign tumors that are generally asymptomatic. Uncertainty still exists surrounding their etiology. Surgical management depends on hormone production, tumor size, high risk features on imaging and patient consent.  Additional information is needed to better understand myelolipomas, their etiology, and clinical management.  Incidentalomas may confuse the physician and patient. Ensuring proper multidisciplinary management based on the clinical guidelines of endocrinology allowed a satisfactory resolution of this case.

## Introduction

The term incidentaloma is derived from “ *incidental tumor,*” describing a mass discovered on imaging by pure chance ^1^. When discussing adrenal incidentalomas (AIs), this refers to a finding of a visible adrenal mass greater than 1cm in diameter found on imaging performed for other medical causes ^2^. In general, adrenal tumors are detected in 0.4% of abdominal ultrasounds and occur with ten times greater frequency in those with a positive cancer history ^3^. Exclusion criteria for AIs include patients who present with manifestations of adrenal dysfunction ^2^ and those with extra-adrenal cancers in the process of stratification ^4^.

Advances in modern diagnostic methods have produced a greater prevalence of AIs, especially due to advances in CT and MRI technology ^3^. Incidental adrenal masses are found in 2–4% of abdominal CT scans and the frequency increases in correlation with the patient’s age - adding 0.2% in the third decade of life, up to 7% in those greater than 70 years old ^4^. Among these, non-functional adenoma remains the most frequent (60–85%), while a minority present as functional adenomas (5–16%) ^5^. Of functional masses, 6% consist of pheochromocytomas, 5% are subclinical Cushing Syndrome, 5% are adrenal carcinoma, 2% prove to be a metastasis, and the rest belong to other etiologies, such as myelolipomas, hematomas, cysts, or lymphomas ^6, 7^.

Nevertheless, the retroperitoneal location increases the difficulty of detection during a standard physical exam. This often leads to the late diagnosis of such tumors only when clinical systemic manifestation is present - in the case of functional incidentalomas - or the compromise of the adjacent tissues secondary to abnormal gland growth ^8^. According to endocrinology guidelines, both hormonal and radiographic evaluation must be performed in order to rule out subclinical states ^9, 10^. In general, masses ≥4cm are removed surgically, independent of functionality. Furthermore, all functional tumors and those with malignant characteristics undergo an adrenalectomy under endocrinologic supervision. Non-functional adenomas, small myelolipomas, and benign asymptomatic cysts do not require surgical intervention ^10^.

With this in mind, providers must remember two primary questions, first asking “Is the mass hormonally active?” as this differentiates between functional and nonfunctional masses ^5, 6^. Additionally, asking “Are there malignant characteristics?” proves equally important. This is determined by the radiologic imaging that look for heterogeneity, poorly delimited borders, the presence of necrosis, hemorrhage, calcification, or an attenuation coefficient greater than 20 Hounsfield Units ^7^.

This case report describes a giant right upper quadrant incidentaloma in an asymptomatic patient that was initially thought to be a hepatic hemangioma, due to its size and location, which was later confirmed to be an adrenal tumor.

## Case Report

A 54 year old asymptomatic female patient was seen by her family physician in Marcaibo, Venezuela, for her annual health exam in January 2014 in a primary care center. She had no complaints, except for recent unintended weight gain. Her past medical and surgical history are notable for a left breast lumpectomy (1973), a salpingectomy (1994), a hysterectomy without oophorectomy for NIC III (2005), and a left unilateral oophorectomy for ovarian torsion (2007). The patient used no medications and has no known allergies, and denied tobacco, alcohol, or drug use. The patient is monogamous and happily married. Her family history is notable for a sister who died of Hodgkin Lymphoma.

On physical exam, the patient was afebrile with normal vital signs. Her weight was 92.5 kg, 1.74 meters tall, with a BMI of 30.6. She appeared well hydrated with moist mucous membranes. She had an unremarkable exam - no findings of violaceous striae, acanthosis, acrochordons, or signs of virilization.

Laboratory results showed a normal complete blood count, mixed dyslipidemia, fasting blood glucose levels >125 mg/dl (normal range, 70–100 mg/dl) on more than two occasions, and HOMA1-IR index >2.5 (normal index, ≤ 2.5) ( Table 1); meeting the diagnostic criteria for type 2 diabetes mellitus (DM2) and metabolic syndrome. Initial recommendations were lifestyle changes, including 30 minutes walks five days a week, and a nutritionist consult. Additionally, pharmacotherapy, sitagliptin/metformin (Janumet ^®^, 50/1000mg) 1 tab daily, ezetimibe/simvastatin (Vytorin ^®^,10/40 mg) 1 tab daily, gemfibrozil (Lipontal ^®^, 900 mg) 1 tab daily, and orlistat (Xerogras ^®^, 120 mg) 1 cap daily, was initiated.

**Table 1.** Results of laboratory exams.

Laboratory	October 2013	November 2013 - January 2014 (initial treatment)	April 2014 (treatment control - pre-operative)
**Cholesterol - Total**	**264,30 mg/dl**	**247 mg/dl**	**121.30 mg/dl**
**HDL - C**	**53 mg/dl**	**41 mg/dl**	**36 mg/dl**
**LDL - C**	**164,64 mg/dl**	**165 mg/dl**	**67.64 mg/dl**
**Triglycerides**	**236 mg/dl**	**204 mg/dl**	**86 mg/dl**
**Creatinine**	**0.6 mg/dl**	**0.7 mg/dl**	**0.8 mg/dl**
**Uric Acid**	**6.9 mg/dl**	**5.0 mg/dl**	**4.2 mg/dl**
**Urea**	**23 mg/dl**	**21 mg/dl**	**29 mg/dl**
**AST**	**19.28 UI/L**	**9 UI/L**	**21.92 UI/L**
**ALT**	**17.00 UI/L**	**10 UI/L**	**21.11 UI/L**
**Blood Glucose**	**148,80 mg/dl**	**129 mg/dl**	**94.35 mg/dl**
**Fasting Insulin**		**11.50 uIU/ml**	
**Postprandial Insulin**		**103.30 uIU/ml**	
*HOMA		**3,5**	

*HOMA-IR = [Basal Insulin (IU/ml) × GA (mg/dL)/405]

**Figure 1.**
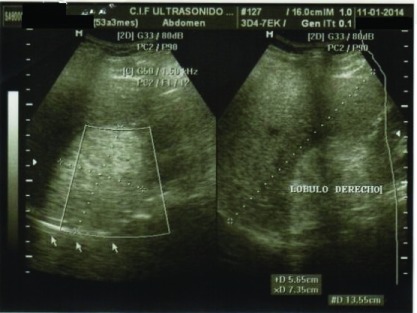
Abdominal ultrasound of the patient. A hyperechogenic 5.6 × 7.3 cm anchor is observed in segment V of the right hepatic lobe suggestive of an incidental hemangioma.

**Figure 2.**
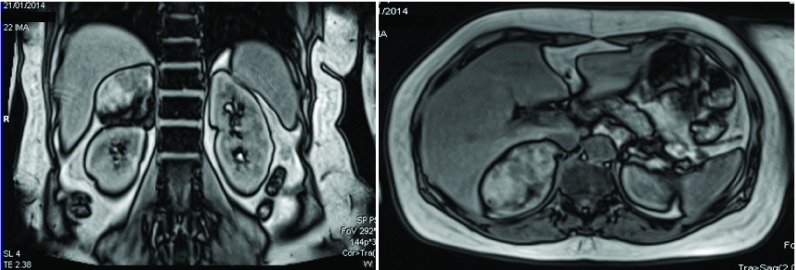
Triphasic abdominal MRI showing a right 7.0 × 6.0 cm adrenal incidentaloma. Left panel, longitudinal cut; right panel, transverse cut. Performed using SIEMENS Magneton Essenza 1.5 TESLA.

Simultaneously, a right upper quadrant ultrasound was ordered showing slight hepatic steatosis, as well as a round space occupying lesion with well-defined hyperechoic borders measuring 5.6×7.3cm in segment V of the right lobe suggestive of a hemangioma. Of note, a bilateral non-obstructive nephrolithiasis was observed ( Figure 1). Due to these findings, the patient was referred to a local hospital diagnostic center for imaging studies, a triphasic hepatic MRI was performed as part of an additional workup. This identified a 7.0×6.0cm right adrenal space occupying lesion suggestive of a large adrenal adenoma ( Figure 2). A hormone profile was performed with normal results - classifying this mass as a non-functional adenoma. Lack of reagents in local laboratories caused that the patients moved to Avila Clinic in Caracas (Capital of Venezuela) ( Table 2). The work up was completed with a serologic evaluation to rule out fungal infection with negative results for mycoplasma IgM (0.15; normal range: 0.00 – 0.90).

**Table 2.** Specific cortical and medullary adrenal hormones (February 2014).

Adrenal cortex			
*Zona reticularis*		*Zona fasciculata*	
Hormone	**Result**	**Hormone**	**Result**
Testosterone - Total	0.09 ng/ml (VN: 0,06-0,82)	Urine cortisol occasional	12.30 ug/dl (VN: 0,20-50,00)
Free Testosterone	0.79 pg/ml (VN: 1,20-6,60)	Cortisol (am)	5.50 ug/dl (VN:5-25)
DHEA-S	76.60 ug/dl (VN: 35,40-256,30)	Cortisol (pm)	4.21 ug/dl
Androstenedione	1,10 ng/nl (VN: 0,85-10,00)		
**Adrenal medulla (in urine)**			
***Catecholamines*** *		***Metanephrines*** **	
Adrenaline	13 mcg/24 hrs (VN: < 20)	Metanephrine/Urine	43.0 mcg/L
Dopamine	706 mcg/24 hrs (VN:<600)	Metanephrine/24 hrs	166.0 mcg/24 hrs (25,0-312,o)
Noradrenaline	10 mcg/24 hrs (VN: < 90)		

24 hr urine collection:* 3.377 ml/24 hrs and **3.860.0 ml/24 hrs

**Figure 3.**
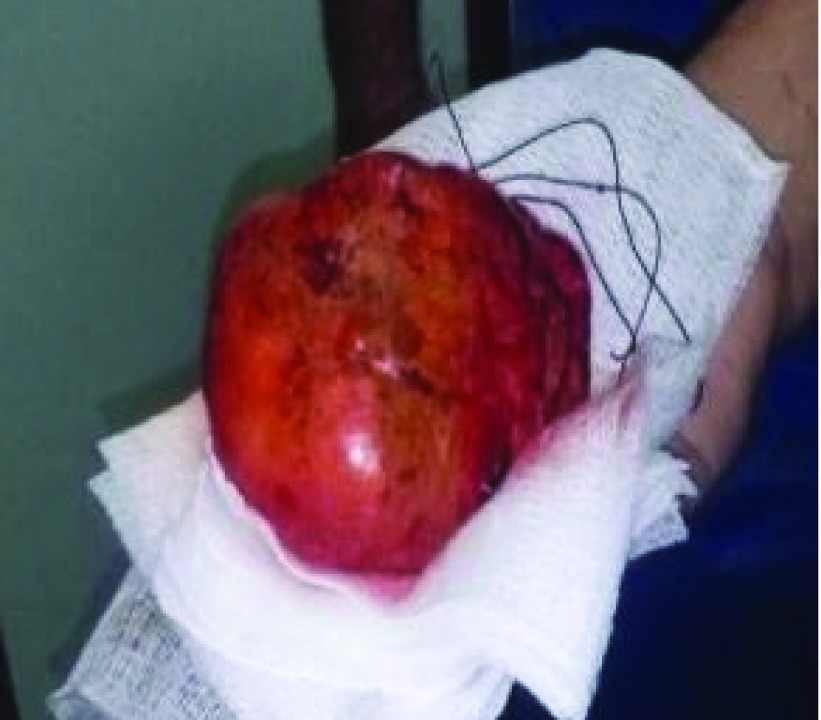
Myelolipoma evaluation. Surgical specimen, macroscopic. Amado Polyclinic, Maracaibo- Edo Zulia (10/04/2013).

In April 2014, a right subcostal adrenalectomy was performed in at a level three hospital so as to ensure the presence of an intensive care unit due to the potential bleeding risk. The pathology report described a 4×7×6cm adrenal mass with a grey-yellow surface covered partially with a thick grey capsule with brown areas with a hemorrhagic and yellow adipose center. The microscopic evaluation showed an external layer of clear cortical cells of the adrenal granulosa; a center made of mature adipocytes and all three hematopoietic cell lines without calcifications or fibrosis. The final diagnosis was determined to be an adrenal myelolipoma ( Figure 3).

The patient experienced no post-surgical complications. She has subsequently completed regular physical activity and continues with the same treatment at the same dosage. Standard laboratory checks at three months showed notable improvement in all parameters.

## Discussion

Adrenal myelolipoma is a rare encapsulated benign tumor described first in 1905 by Gierke ^11^ and later named by the French pathologist Charles Oberling in 1929 ^12, 13^. These tumors are metabolically inactive - or nonfunctional - and composed of adipose and hematopoietic cells originating from the adrenal stroma. They are predominantly asymptomatic and tend to be discovered incidentally ^13– 15^.

The incidence of these tumors is between 0.08–0.4% ^12^, although they comprise 15% of the AIs discovered due to advances in radiographic anchorry ^13^. They frequently present between the fifth and seventh decades of life without a predominance in either sex - though there is a greater incidence in the right adrenal gland ^15^. Though the adrenal location predominates, there have been discoveries in other locations with a preference for the presacral region, and less frequently in gastric, hepatic, ganglionic lymphatics, cranium, and spleen locations ^16^. These statistics are in accordance with this case report.

The etiology for adrenal myelolipoma is not clear with numerous theories being proposed. Some suggest a metaplasia of the adrenal and myeloid cells that migrated during embryogenesis, extramedullary hematopoiesis, and embolization of osseous medulla elements ^17^. This metaplasia may occur as a response to necrosis, stress, infections, or prolonged adrenocorticotropic hormone (ACTH) stimulation ^11, 18^. For example, Al-Bahri *et al.* ^19^ reported a case of a large bilateral myelolipoma in a 39 year old male with a history of congenital adrenal hyperplasia secondary to a 21-α hydroxylase deficiency treated with steroids starting in childhood. This was later stopped during adolescence with a subsequent myelolipoma development - supporting the theory that ACTH stimulation causes adrenocortical metaplasia. Finally, giant myelolipomas usually are associated with hematologic disorders, like hereditary spherocytosis, thalassemia, and falciform anemia, as a response to adrenal stimulation from erythropoietin ^20^.

Recent cytogenetic analyses propose that myelolipomas are out-of-place masses of myeloid cells. Mitsui *et al*. described an extremely rare case with the presence of osseous tissue with cells similar to osteoblasts ^21^. Upon immunohistochemical analysis, there were positive results for bone morphogenetic protein 2 (BMP2), which acts as an inductor for osseous formation and the β-catenin that intervenes in the signal pathway. This finding can help give insight into myelolipoma tumorigenesis.

Researchers have also identified (3,21)(q25;p11) chromosomal translocations in patients with myelolipomas and hematological neoplasias ^18^. Because of this, some consider myelolipomas as variants of multiple endocrine neoplasias ^22^, while others recommend that they be grouped with other tumors, such as lipomas, teratomas, liposarcomas, or angiomyolipomas ^23, 24^. Despite its benign characteristics, the pathological studies and immunohistochemical evaluation (not performed due to lack of reagents) was recommended, because of the patient's personal and family history that increased risk for malignant results.

Though these tumors are nonfunctional ^13– 15, 25^, there may be the coexistence of myelolipoma with hyperplasia in any of the three adrenal cortical zones ^26, 27^. For these cases, treatment is adrenalectomy (just as in any case of myelolipomas >6cm) independent of its functionality, due to the risk of intratumoral necrosis, hemorrhage from rupture or compression of adjacent structures due to mass effect ^28^. Alternatively, nonfunctional tumors ≤4cm with benign characteristics are recommended to be periodically monitored with radiological and biochemical evaluations. For masses between 4 and 6cm, the surgical intervention should be based on presenting characteristics, growth rate, and the patient’s preference ^7, 29^.

It is estimated that 20% of AIs will have subclinical hormone production and these patients represent an at-risk population with greater risk of metabolic disorders and cardiovascular disease ^7, 19^. In the present case, the patient’s hormone values were within normal parameters - ruling out subclinical states, including Conn's Syndrome (hyperaldosteronism), Cushing Syndrome or pheochromocytoma. Nevertheless, the presence of myelolipoma is associated with obesity, DM2 and dyslipidemia warranting pharmacological intervention ^30^. This was further emphasized through a retrospective review of 34 AIs in patients of both sexes over the age of 50, where over half suffered from hypertension, 20.6% had DM2, and 37% had obesity. Of these, 80% were histopathologically confirmed to be adenomas with one being a myelolipoma ^25, 30^.

As strengths, we can point out the collaboration between different levels of medical attention and the shared effort of the family and the patient to travel to another state to complete this medical Care. Despite the Venezuelan medical assistance crisis, a relatively quick resolution of the case was achieved. Lastly, we emphasize the compliance with the protocol for proper management of adrenal tumors.

The limitations include the inability to perform the hormonal profile and determine whether the tumor was functional or not. Additionally, the choice of imaging could have been better. Specifically, the use of MRI instead of CT is not the first choice for the diagnosis of the myelolipoma; however, this occurred because the initial diagnosis was directed towards a hepatic hemangioma.

## Conclusions

Adrenal myelolipomas are rare benign tumors that are generally asymptomatic, whose size ranges from a few millimeters to over a dozen centimetres. Much uncertainty exists surrounding the etiology of these masses with continued debate in the current literature on whether or not they are true neoplasms or manifestations secondary to a reactive process ^26^. In general, surgical management depends on hormone production, tumor size, high risk features on imaging and patient consent. Yet additional studies and information are needed to better understand myelolipomas, their etiology, and clinical management.

Lastly, this case demonstrates how family physicians can manage various aspects of patient care through the facilitation of medical treatments, surgical interventions, and ensuring a proper multidisciplinary approach based on the endocrinology clinical guidelines.

## Consent statement

Written informed consent was obtained from the patient for the publication of the patient’s details and accompanying anchors.
