# Supplementary material for: Case Report: An incidentaloma that catches your eye - adrenal myelolipoma
Source: F1000Res. 2017 Jul 18;6:1140. [Version 1] doi: 10.12688/f1000research.11766.1 (PMC5538031; doi:10.12688/f1000research.11766.1)
Supplement: Peer review history

# Peer review: Referee response for version 1

**DOI:** 10.5256/f1000research.12710.r24335

**Published:** 2017-07-31

**Authors:** Ana Nunes Barata

**Affiliations:** 1 ACES Amadora, UCSP Buraca , Lisbon, Portugal

This case provides an extensive and comprehensive background and description of a case of an adrenal myelolipoma. As this is not a common condition, sharing the knowledge on a rare case may facilitate other practitioners to reach the correct diagnosis. It also includes description of relevant differential diagnosis as well as treatment.

I have read this submission. I believe that I have an appropriate level of expertise to confirm that it is of an acceptable scientific standard.

# Peer review: Referee response for version 1

**DOI:** 10.5256/f1000research.12710.r24338

**Published:** 2017-07-25

**Authors:** José Manuel Ramírez Aranda, Jesús Zacarías Villarreal- Perez

**Affiliations:** 1 School of Medicine, Family Medicine Department, Autonomous University of Nuevo Leon, Monterrey, Mexico; 2 School of Medicine, Endocrinology Department, Autonomous University of Nuevo Leon, Monterrey, Mexico

I think the case report fulfills diagnostic criteria for Adrenal Incidentaloma as Kim and Zeiger *et al.* rightfully have pointed it out. The clinical case was well studied according to accepted guidelines. I consider that the manuscript is well written. Perhaps the only thing that would call my attention is in the Discussion section since it seems to me unnecessarily extensive, but it depends on journal requirements and policies.

 One suggestion in the abstract: Use Arabic number to describe the type of Diabetes.

We have read this submission. We believe that we have an appropriate level of expertise to confirm that it is of an acceptable scientific standard.
